# Iatrogenic Venous Compression Syndrome Following Bilateral Hip Arthroplasty: A Unique Case of Bilateral Femoral Vein Compression in a Patient With May-Thurner Syndrome

**DOI:** 10.7759/cureus.56362

**Published:** 2024-03-18

**Authors:** Roel Meeus, John S Christian, Nele Meeus, M. Akram Khan

**Affiliations:** 1 Cardiology, Catholic University of Leuven, Leuven, BEL; 2 Cardiology, Cardiac Center of Texas, McKinney, USA

**Keywords:** venous insufficiency disorders, pelvic congestion syndrome, bilateral iatrogenic venous compression, iliac vein compression, may-thurner's syndrome, case reports

## Abstract

Iatrogenic venous compression syndrome is defined by extrinsic vein compression due to medical hardware, particularly relevant after joint replacement surgeries. Inserting medical hardware can lead to immediate risks such as deep vein thrombosis and pulmonary embolisms due to local tissue inflammation. The long-term issues include venous insufficiency due to chronic vessel irritation, subsequently causing intimal proliferation and thickening. Despite the existing knowledge of venous compression syndromes, iatrogenic cases are severely underreported. Here, we present a unique case of bilateral common femoral vein compression in a patient with May-Thurner syndrome and prior bilateral hip arthroplasty.

An 85-year-old man with a history of venous insufficiency and bilateral hip arthroplasty for osteoarthritis presented with bilateral leg edema. Unsuccessful sclerotherapy and radiofrequency ablation led to a referral to a vascular specialist for venous duplex scans, venograms, and intravascular ultrasound.

May-Thurner syndrome was revealed in the left common iliac vein, prompting the deployment of an 18 mm × 16 mm stent. Subsequently, during a venogram, what initially appeared to be a vasospasm in the left common femoral vein was diagnosed as extrinsic iatrogenic venous compression due to acetabular hip screws. This was found after two IV injections of 400 mg nitrogen and one balloon angioplasty could not resolve the compression. After advancement over a 0.35" microwire and accurate positioning over the center of the left common femoral vein lesion, a 16 mm × 90 mm stent was deployed. The venogram and intravascular ultrasound also showed a similar compression in the right common femoral vein. Another 400 mg IV nitrogen did not expand the lesion, so it was concluded that there was similarly an iatrogenic venous compression of the right common femoral vein, also due to acetabular hip screws in the right femur. A follow-up was scheduled a couple of weeks later to address the issue in the right common femoral vein.

The underreported issue of iatrogenic venous compression following joint replacements highlights the need for better recognition and management of vascular complications due to inflammation and intimal proliferation. This is especially the case in high-risk patients, such as those with May-Thurner syndrome.

## Introduction

Iatrogenic venous compression syndrome, defined by extrinsic vein compression, is a complex clinical disorder with diverse causes. It can be caused by any medical operation inadvertently, but it is often associated with orthopedic and vascular surgery [1-3]. Iatrogenic venous compression is linked to an increased incidence of deep venous thrombosis (DVT), as well as venous insufficiency [4]. The proximity of medical hardware, such as trans-acetabular screws, can cause local bleeding. This results in tissue inflammation and iatrogenic compression of neighboring veins. The link between this compression and an elevated risk of DVT is described by Virchow's triad, which includes endothelial damage, hypercoagulability, and venous stasis. Veins are easily compressed and are prone to stasis and thrombosis due to their thin walls and low pressure [5]. Venous insufficiency is a less common complication of iatrogenic venous compression syndromes. Its presumed pathophysiology is a prolonged presence of medical hardware and its pressure on the vein causing chronic endothelial irritation, intimal proliferation, and progressive narrowing of the vessel, ultimately resulting in significant stenosis [1]. This gradual process explains why most patients experience venous insufficiency symptoms only after a few years following surgery [1].

Iatrogenic venous compression syndromes are a subset of iliofemoral venous compression syndromes. They refer to a group of disorders defined by venous structural compression, which is often caused by anatomical, disease-related, or iatrogenic causes.

The May-Thurner syndrome represents a noteworthy anatomical variant, involving the compression of the left common iliac vein (LCIV) by the right common iliac artery [6]. Similarly, conditions such as the Nutcracker syndrome and Paget-Schroetter syndrome contribute to venous compression owing to distinct anatomical anomalies [6]. Disease-induced variants are evident in instances where enlarged cysts, tumors, and abdominal aneurysms impose compression upon venous structures [7].

In addition to anatomical and disease-related factors, iatrogenic venous compression syndromes have been recognized as a consequence of surgical interventions. However, the precise prevalence of these hardware-related occurrences remains indeterminate, and their documentation is often limited. While iatrogenic venous compression syndromes stemming from surgical interventions are likely infrequent [2,3], they contribute to the broader spectrum of iliofemoral venous compression syndromes.

In this article, we present a unique case in which a patient, in addition to being diagnosed with May-Thurner syndrome, was also diagnosed with a bilateral iatrogenic venous compression, affecting both the right and left common femoral veins, due to prior total hip arthroplasty.

This article was previously presented as a podium presentation at the 2023 VEITHsymposium on November 17, 2023.

## Case presentation

An 85-year-old Caucasian male patient with a history of venous insufficiency and prior bilateral total hip arthroplasty due to osteoarthritis, presented with bilateral swollen, heavy, and fatigued legs. He complained of being slowed down and prevented in his mobility, despite recently undergoing unsuccessful sclerotherapy and endovenous radiofrequency ablation treatment of the left and right greater saphenous veins. These interventions provided him no significant improvement in symptoms as he still experienced them daily.

In June 2023, due to the persistence and exacerbation of symptoms despite prior interventions, the patient was referred for a secondary consultation from a vascular specialist. Upon thorough physical examination, notable findings included evident swelling in the extremities, the presence of varicose veins in the left lower extremity, bilateral telangiectasias, evident hemosiderin staining, and 2+ edema observed on both the right and left sides. Bilateral pitting edema was also documented, while signs of erythema, ecchymosis, and open ulcers were conspicuously absent. Subsequent to a venous duplex ultrasound, the patient received a diagnosis of class III varicose veins, classified according to the Clinical, Etiology, Anatomic, Pathophysiology (CEAP) classification system for venous disorders. Despite the application of conservative treatments and the implementation of endovenous radiofrequency ablation on both the left and right greater saphenous veins in the lower extremities, the patient experienced no alleviation of symptoms. In light of this, and with the patient's and family's concurrence, a decision was made to pursue further comprehensive medical assessments to elucidate the underlying causative factors. A follow-up plan was established, with a venogram scheduled to provide continued insight into the condition's progression and inform subsequent steps in the management process.

**Figure 1:**
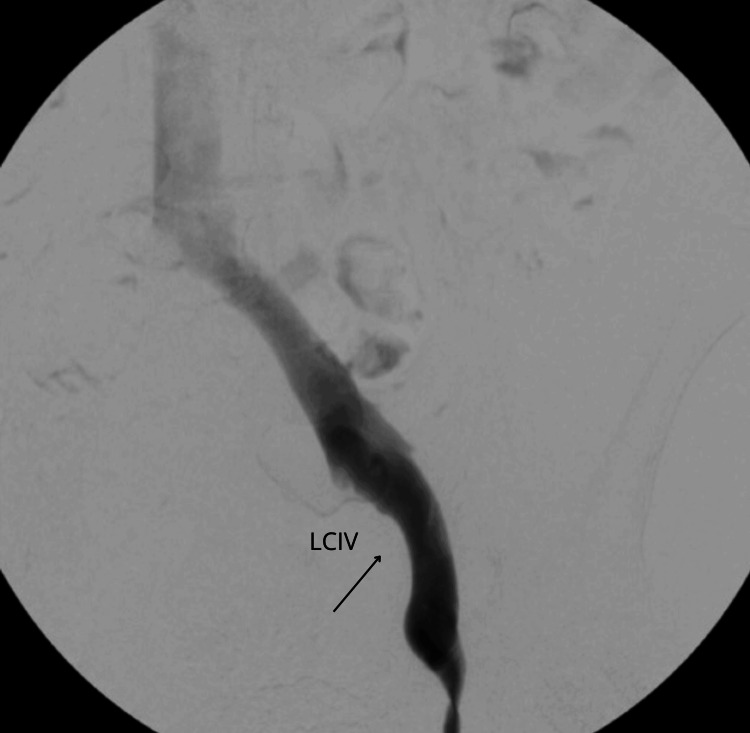
Visualization of the compression of the left common iliac vein (LCIV).

In August 2023, the patient underwent a comprehensive medical procedure involving a venogram and an intravascular ultrasound (IVUS) assessment, conducted with the aim of excluding the presence of May-Thurner syndrome. The procedure involved gaining venous access through the right femoral vein, followed by the careful advancement of a 0.35" microwire in a retrograde manner to reach the LCIV. Contrast material was subsequently introduced into the system to achieve visualization of the LCIV. The imaging revealed a mild constriction within the left common femoral artery at the lumbar level L5 (Figure 1).

Following this, an IVUS catheter was meticulously guided to the targeted area, enabling detailed visualization of the iliac vein. The IVUS examination confirmed the compression of the LCIV, definitively corroborating the diagnosis of May-Thurner syndrome.

Upon ensuring precise placement, an appropriately sized stent measuring 18 mm × 16 mm was introduced over the previously positioned microwire. The subsequent phase of the procedure ensued, involving the continued advancement of the microwire to achieve deeper penetration, contrast was given to facilitate visualization of the left common femoral vein. The venogram images revealed a conspicuously irregular venous compression, anatomically situated at the level of the femoral head. Initially, the working diagnosis leaned toward vasospasm, and an intervention was initiated by administering a 400mg intravenous dose of nitrogen with the intention of mitigating the presumed vasospasm. Following this, the contrast was reintroduced into the system, yet the site of compression displayed no improvement. Consequently, a second dose of 400 mg nitrogen intravenous infusion was administered. Despite this effort, the contrast-enhanced visualization exhibited persistent unaltered findings. Subsequently, balloon angioplasty was undertaken as an attempt to alleviate the venous compression. Regrettably, this intervention also yielded no improvement in the observed condition. After a comprehensive assessment, a definitive conclusion was drawn: the venous compression that had initially been speculated to be a vasospasm of the left common femoral vein due to the presence of the catheter, was, in fact, a genuine stenosis induced by external venous compression.

An IVUS examination revealed no calcifications within the lesion, suggesting iatrogenic venous compression syndrome due to chronic irritation by an adjacent acetabular screw, leading to significant stenosis. This inference was supported by the patient's history of total hip replacement surgery.

**Figure 2:**
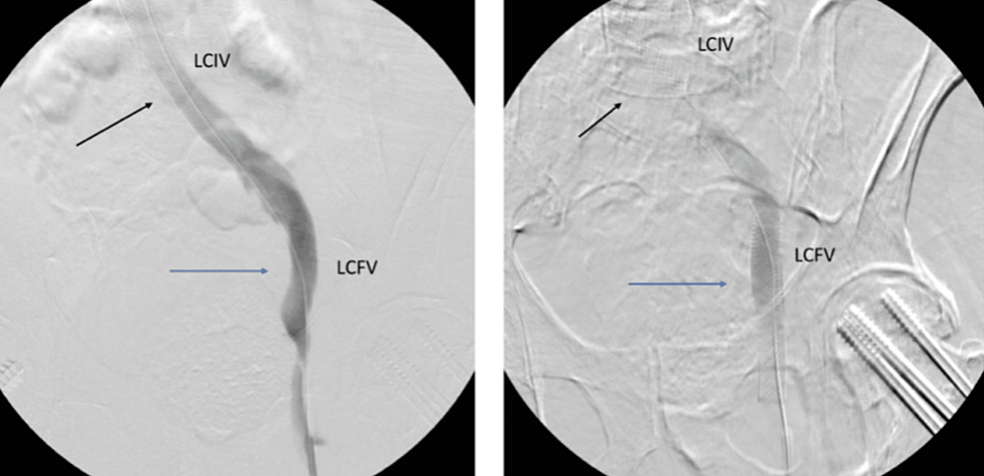
Visualization of the left common femoral vein (LCFV) (blue) before and after stent placement with close proximity of the acetabular hip screw. The stent placed in the left common iliac vein (LCIV) for the May-Thurner syndrome is also visible (black).

With precise positioning achieved within the left common femoral vein, a stent measuring 16 mm × 90mm (Venous WALLSTENT, Boston Scientific, Natick, Massachusetts, USA) was meticulously introduced to alleviate the extrinsic venous compression (Figure 2).

**Figure 3:**
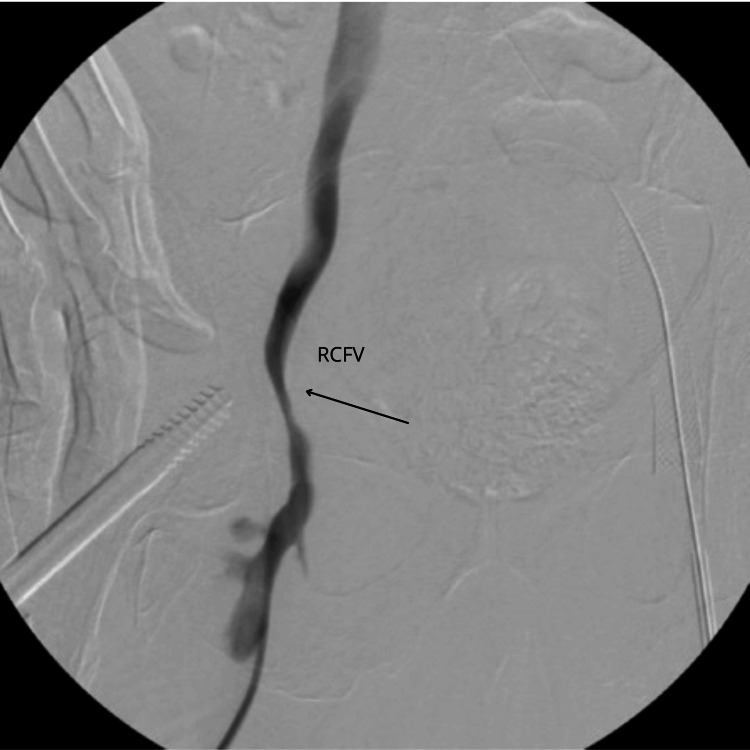
Visualization of the compression of the right common femoral vein (RCFV) with narrow proximity of the right acetabular hip screw.

Subsequently, contrast injection was administered to facilitate the visualization of the right common femoral vein. Both venogram and IVUS examinations unveiled a significant, irregularly shaped compression within the right common femoral vein, situated anatomically at the level of the right femoral head. This compression was notably accentuated by the immediate presence of hardware. Initial evaluation led to the presumption that this condition, akin to the prior instance in the left common femoral vein, was attributable to vasospasm. In an endeavor to counteract this, a dosage of 400 mg of intravenous nitrogen was administered. However, as in the previous case, this intervention yielded no improvement in the observed condition (Figure 3).

Based on the previous experience, a diagnosis of iatrogenic venous compression syndrome in the right common femoral artery was made, also due to acetabular screws in the femoral head causing significant stenosis. A follow-up appointment was scheduled for a venogram procedure aimed at stent placement to alleviate the observed compression.

## Discussion

Chronic venous insufficiency (CVI) occurs when veins in the lower extremities fail to guide blood back to the heart effectively. This can result in persistently high pressure in the veins and lead to symptoms such as tightness, heaviness, fatigue, leg cramps, restless legs, and skin changes such as thickening or discoloration. The most common reasons for CVI include a lack of functional valves in the lower limb veins from birth, biochemical alterations in the venous valves, and DVT. Risk factors include obesity, smoking, pregnancy, sedentary lifestyle, hypertension, and history of DVT. Complications of CVI include venous ulcers, thrombophlebitis, DVT, pulmonary embolism, bleeding, secondary lymphedema, and chronic pain [8-10]. In most cases, complaints of CVI can be approached with compression stockings, endovenous radiofrequency ablation, or sclerotherapy [9]. If the patient doesn’t experience alleviation of his symptoms, the problem is more likely situated above the level of the groin, where more rare syndromes such as May-Thurner syndrome, Nutcracker syndrome or similar pathologies should be considered [11]. In this case, perforating acetabular hip screws caused an inflammatory reaction with fibrosis, leading to chronic endothelial irritation and subsequent stenosis.

Perforations associated with the use of periacetabular screws in total hip arthroplasty have been reported at rates ranging from 0.9% to 7.0%, as documented by Eberl et al [12]. These occurrences, while not rare, often lack clinical significance. Consequently, most experts do not advocate for repositioning the screws in such cases. While the literature on iatrogenic venous compression due to acetabular screws remains relatively scarce, there have been reports detailing severe adverse events, including acute right leg DVT and massive iliofemoral thrombosis [13]. Additionally, cases of venous insufficiency surfacing several years after total hip arthroplasty have been documented [14]. However, it is worth noting that most documented cases involving acetabular screw incidents predominantly revolve around DVT and thrombosis [5]. CVI has garnered more extensive attention as a potential consequence of iatrogenic venous compression. It has been more widely reported as an outcome of anterior pedicle screw perforation in spinal fusion surgeries and scoliosis correction procedures [1]. Furthermore, case reports have detailed venous compression as a complication in vascular repairs [2,3] and even in penile prosthesis surgeries [5].

However, what sets our case apart from most reports is its unique presentation of bilateral pathology. To the best of our knowledge, this is the first documented case reporting bilateral iatrogenic compression resulting from bilateral total hip arthroplasty. In this particular patient, an underlying May-Thurner syndrome likely played a catalytic role. This pre-existing condition predisposed him to developing such complications. It is noteworthy that both May-Thurner syndrome and iatrogenic venous compression are encompassed within the broader spectrum of iliofemoral venous compression syndromes, sharing a common pathogenesis.

The continuous pressure exerted by the acetabular screws led to prolonged endothelial irritation, fostering intimal thickening and stenosis [1]. Consequently, this pathophysiological process contributed to the aggravation of venous insufficiency symptoms several years post-surgery. In this particular case, the placement of stents was deemed the most effective intervention for rectifying the stenosis and restoring adequate vascular flow.

As discussed, iatrogenic venous compression syndromes constitute a subset within a broader spectrum of venous compression syndromes. This overarching category encompasses anatomical variations such as May-Thurner, Paget-Schroetter, and Nutcracker syndromes [6], as well as disease-related variants like those associated with enlarged cysts and abdominal aneurysms [7]. While anatomical and disease-related variants have garnered substantial attention in medical literature, iatrogenic venous compression remains a significantly underdiagnosed and underreported contributor to venous insufficiency.

A comprehensive understanding of vascular anatomy, coupled with meticulous pre- and intraoperative imaging assessments of vessels vulnerable to compression during hip surgeries, can play a pivotal role in mitigating the incidence of perforations. This proactive approach not only aids in averting complications such as DVT and venous insufficiency, it also facilitates early recognition and management of iatrogenic venous compression syndromes, fostering improved patient outcomes.

## Conclusions

This case highlights the importance of recognizing iatrogenic venous compression syndromes post-joint replacement surgeries, such as total hip arthroplasty. Significant occlusion in both common femoral veins, occurring years after the procedure, underscores the need for long-term monitoring and proactive management. The problem likely stems from hardware proximity, leading to intimal thickening and stenosis. Further research is needed to understand risk factors and preventive measures. Vigilant long-term monitoring is crucial to mitigate potential vascular complications.
